# Intractable Neuropathic Pain in COVID-19-Induced Guillain-Barré Syndrome: A Case Report

**DOI:** 10.7759/cureus.36426

**Published:** 2023-03-20

**Authors:** Stephen Ritter, Daniel Gable, Andrew May, Yousef Darwish, Tracy Friedlander

**Affiliations:** 1 Physical Medicine and Rehabilitation, Johns Hopkins University School of Medicine, Baltimore, USA

**Keywords:** allodynia, miller-fisher syndrome, covid 19, guillain barrè syndrome, neuropathic pain

## Abstract

There is limited literature specific to neuropathic pain in coronavirus disease 2019 (COVID-19)-induced acute inflammatory demyelinating polyneuropathy (AIDP). We present a unique case of a 20-year-old vaccinated female with a past medical history of chronic hepatitis B virus and untreated anxiety who presented to the emergency department due to an intractable headache and horizontal diplopia in the setting of active COVID-19 infection. During acute hospitalization, the patient was diagnosed with the Miller-Fisher variant of Guillain-Barré syndrome (GBS), a disease with a known association with COVID-19. While in the ICU, the patient developed severe, 10/10-rated, distal, symmetric burning pain with associated allodynia requiring a multimodal regimen with combinations of intravenous narcotics, neuropathic medications, topical agents, and desensitization training to attempt to control her pain. Rehabilitation psychology was consulted while she was in chronic ventilatory rehabilitation for supplementation of behavioral pain management strategies with pharmacological approaches for continued pain. After several months and completion of a comprehensive inpatient rehabilitation program, the patient was weaned off intravenous narcotics and prescribed oral pain medications. This patient had the optimal response to amitriptyline, which likely aided in the co-treatment of psychological manifestations of COVID-19 and prolonged hospitalization. This study highlights the pathogenicity of COVID-19-induced AIDP, its potential severity, and the importance of a multidisciplinary approach to managing it.

## Introduction

We discuss a unique case of a young female diagnosed with coronavirus disease 2019 (COVID-19)-induced Miller-Fisher syndrome that resulted in severe neuropathic pain. The neurological manifestations of COVID-19 are well-documented [1]. However, the pathogenesis of chronic pain syndromes associated with these neurological sequelae is not well understood [1]. Neuropathic pain in particular is a common manifestation of both acute inflammatory demyelinating polyneuropathy (AIDP) and COVID-19 [2]. The pathogenesis of neuropathic pain in AIDP is not well described but is hypothesized to be related to the impairment of small nociceptive fibers [2]. Theories on chronic pain related to COVID-19 include viral-mediated effects, autoimmune effects, psychological stressors, and prolonged critical care [1]. There are scarce data in the literature regarding COVID-19-induced AIDP in particular. Our patient's pain was described as persistent despite treatment with mainstay neuropathic pharmaceutical agents including gabapentinoids, antidepressants, tricyclic antidepressants, tramadol, and topical agents [1]. This led to a delay in transferring the patient to inpatient rehabilitation and impacted her participation in rehabilitation. This case highlights the potential severity of this pain syndrome and the importance of a multimodal approach to treatment.

## Case presentation

A 20-year-old vaccinated female with a medical history of chronic hepatitis B virus infection and untreated anxiety presented to the emergency department with a five-day history of intractable headache, horizontal diplopia, and myalgias in the setting of active COVID-19 infection. Imaging and diagnostic studies, including complete blood count, comprehensive metabolic panel, MRI, CT, blood cultures, CSF cultures, and autoimmune antibody panels, were unremarkable. Cerebrospinal fluid (CSF) studies showed a degree of albuminocytological dissociation with highly elevated CSF protein (441 mg/dl), minimal elevation of CSF white blood cells (18 cubic mm), and normal CSF glucose, indicating AIDP. Nerve conduction studies were performed and the only obtainable responses were a normal sural response and severely reduced ulnar and facial motor responses with prolonged distal latencies (right ulnar latency: 5.5 ms, right ulnar amplitude: 1.0 mV, right facial latency: 6.9 ms, right facial amplitude: 0.3 mV). Needle electromyography revealed no abnormal spontaneous activity or detectable voluntary motor responses. These findings were significant for severe sensorimotor neuropathy, which is consistent with AIDP. Despite treatment with intravenous immunoglobulin for AIDP, the patient developed areflexia, bilateral oculomotor nerve palsies, bilateral facial nerve palsies, and quadriplegia. Given her clinical findings, the patient was diagnosed with the Miller-Fisher variant of Guillain-Barré syndrome (GBS), a disease with a known association with COVID-19 [3]. Due to her worsening neurological status, the patient was electively intubated and she required mechanical ventilation for 52 days.

During her acute hospitalization in the ICU, the patient developed severe, 10/10-rated, distal, symmetric burning pain with associated allodynia requiring a multimodal approach and desensitization training. She received combinations of ketamine infusions, intravenous hydromorphone, fentanyl, duloxetine, gabapentin, pregabalin, amitriptyline, acetaminophen, topical agents, and clonazepam. The patient did not respond to a regimen that included gabapentin 1,200 mg every eight hours. Pregabalin was attempted with 200 mg three times daily but this was also unsuccessful. Allodynia made any mobilization extremely difficult and was most severe in her feet and hands, where essentially any cutaneous touch was intolerable. Rehabilitation psychology was consulted after she was transferred to chronic ventilatory rehabilitation and began behavioral pain management strategies to supplement pharmacological approaches for her continued 10/10-rated pain. The rehabilitation psychology team instituted a cognitive-behavioral-based approach to pain management. This approach was multi-faceted and consisted of (1) relaxation strategies (e.g., mindfulness, breathing exercises, guided imagery, and progressive muscle relaxation) to help calm the mind and relax the body; (2) education about pain, to help differentiate hurt from harm and better understand the relationship between thoughts, emotions, behaviors, and pain; and (3) distraction, which can be helpful, in the moment, to divert attention away from pain. Additionally, motivational interviewing techniques were employed to increase engagement in physical and occupational therapy. Rehabilitation psychology also frequently co-treated with rehabilitation therapies to help reinforce these strategies, in real-time, while providing support to the patient and team. This multimodal approach was helpful in managing her pain by increasing feelings of self-efficacy and reducing fear avoidance during rehabilitation. 

After several months and completion of a comprehensive inpatient rehabilitation program, the patient was successfully weaned off intravenous narcotics and started on oral amitriptyline 75 mg nightly, hydroxyzine 25 mg three times daily, and tramadol 50 mg twice daily. The patient had the best response to amitriptyline, as evidenced by the worsening of pain during attempts to wean it off. It also provided the additional benefits in combination with ongoing follow-up with rehabilitation psychology, of treating her anxiety, symptoms of post-intensive care syndrome, and insomnia resulting from prolonged hospitalization.

## Discussion

While neuropathic pain in GBS seems to be related to the impairment of small nociceptive fibers, the pathophysiology of neuropathic pain following COVID-19 is not well understood [1,3]. COVID-19’s unique profile allows it to act on the nervous system either directly or indirectly [1]. Furthermore, it may involve the central or peripheral nervous system [1]. It has been hypothesized that COVID-19-induced neuropathic pain may involve an autoimmune phenomenon between viral epitopes and host antigens [4]. This can create a virus-mediated cytokine storm that may play a role in neuropathic pain after severe COVID-19 illness [1,5]. One retrospective study has reported that up to 25% of hospitalized COVID-19 survivors suffered from neuropathic pain [6]. Notably, anxiety levels were also associated with the presence of neuropathic pain in hospitalized COVID-19 survivors [6]. Neuropathic pain has also been observed in patients after undergoing prolonged critical care [1]. While neuropathic pain has been studied in patients after COVID-19 infection and GBS, there is little research specifically regarding pain in COVID-19-induced GBS [1,3,7]. This study is limited to only one case but involves a young female patient, in contrast to studies with a predominance of older male patients with COVID-19-induced GBS [8].

## Conclusions

It is likely that AIDP was the primary etiology behind our patient’s pain even though she had additional plausible risk factors including COVID-19 infection and prolonged critical illness. These along with untreated psychological disorders may have produced an additive or synergistic effect. The use of other topical, antiepileptic agents, or nonpharmacological techniques could have also been attempted but were not trialed in this particular patient. Given the multifactorial nature of the pain, a combination of pharmacological agents and behavioral pain management strategies should be implemented in these patients.
